# Dynamic and Full-Time Acquisition Technology and Method of Ice Data of Yellow River

**DOI:** 10.3390/s22010176

**Published:** 2021-12-28

**Authors:** Yu Deng, Chunjiang Li, Zhijun Li, Baosen Zhang

**Affiliations:** 1 Yellow River Institute of Hydraulic Research, Zhengzhou 450003, China; dengyu@hky.yrcc.gov.cn; 2 Research Center on Levee Safety Disaster Prevention, MWR, Zhengzhou 450003, China; 3 State Key Laboratory of Coastal and Offshore Engineering, Dalian University of Technology, Dalian 116024, China; lichunjiang0405@mail.dlut.edu.cn (C.L.); lizhijun@dlut.edu.cn (Z.L.)

**Keywords:** river ice, remote monitoring, air-coupled radar, temperature, flow ice density and velocity, ice thickness, water level, Yellow River

## Abstract

Regarding the ice periods of the Yellow River, it is difficult to obtain ice data information. To effectively grasp the ice evolution process in the ice periods of the typical reach of the Yellow River, a fixed-point air-coupled radar remote monitoring device is proposed in this paper. The device is mainly composed of an air-coupled radar ice thickness measurement sensor, radar water level measurement sensor, temperature measurement sensor, high-definition infrared night vision instrument, remote switch control, telemetry communication machine, solar and wind power supply, lightning protection, and slewing arm steel tower. The integrated monitoring device can monitor ice thickness, water level, air temperature, ice surface temperature, and other related parameters in real time. At present, devices have obtained the ice change process of fixed points in ice periods from 2020 to 2021. Through a comparison with manual data, the mean error of the monitoring results of the water level and ice thickness was approximately 1 cm. The device realizes the real-time monitoring of ice thickness and water level change in the whole cycle at the fixed position. Through video monitoring, it can take pictures and videos regularly and realize the connection between the visual river and monitoring data. The research results provide a new model and new technology for hydrological monitoring in the ice periods of the Yellow River, which has broad application prospects.

## 1. Introduction

The ice disaster of the Yellow River is one of the important natural disasters in the winter and spring seasons from China. According to historical statistics, the ice disaster of Yellow River nearly occurred 30 years ago from 1855 to 1955. Since 1986, there have been 13 ice flood break levees in the Inner Mongolia section of the Yellow River, which have caused huge economic losses and social impact. The Inner Mongolia reach of the Yellow River undergoes different degrees of ice disasters in winter and spring every year, which are affected by the special geographical location and climate conditions [1,2,3]. Due to the lack of a research foundation on the occurrence and development mechanism of ice floods in the Inner Mongolia reach [4,5,6,7], the large range of ice jam and ice dam disasters, the short forecast period, and the difficulty in obtaining ice information, ice disaster prevention measures are greatly passive [8,9,10,11]. Therefore, it is urgently necessary to carry out research on field monitoring technology of Yellow River ice.

Around the 1980s, scholars worldwide carried out field observations of river ice regimes. Parkinson [12] and Marsh [13] effectively observed the water temperature under the ice cover during the freezing period and during the river breakup period, and Gerard [14] observed the cracking process of river ice. In recent years, to ensure the personal safety of observers, some new observation technologies have emerged. For example, Ford et al. [15] used an ice plug thickness meter to observe the ice thickness, Morse et al. [16] used the Acoustic Doppler Current Profiler (ADCP) to measure the floating ice velocity and ice cover thickness at specific points, and Jasek et al. [17] used a SWIPS system to measure the ice sheet growth and ice melting speed.

In view of the ice observations of the Yellow River, the manual ice observation technology is mainly traditional ice observation technology. For example, artificial perforation and ice rulers have been used for the measurement of ice thickness. This method not only has low efficiency, but also has a limited measurement area, which makes it impossible to realize real-time observations. With the development of new technology, different physical methods, such as acoustic waves, electromagnetic waves, and resistivity are emerging to measure ice, which can obtain a large amount of data in a short time without damaging the ice layer [18,19,20,21,22,23]. The resistivity method mainly takes advantage of the difference of electrical conductivity of different agents to measure ice thickness. Qin Jianmin et al. [24,25,26] realized the collection of ice and water regimes based on the difference of conductivity of air, ice, and water with the resistivity method, but this kind of contact equipment is easily damaged by static ice extrusion and ice impact. The geoelectric ice test method takes advantage of the difference between the high-frequency curve part and the low-frequency curve part of the electrical curve to invert the flow ice in the river. The method has strong real-time performance, relatively low monitoring cost, and high accuracy, but it can only obtain the parameter information of local river ice and does not provide visual images of ice [27,28]. Satellites or aerial remote sensing monitoring methods have a large observation range and wide coverage. However, due to the long revisit period, low remote sensing resolution, and vulnerability to the interference of atmospheric clouds, the ability to grasp the local details of the Yellow River is not strong, and the real-time performance cannot meet the needs [29,30]. In addition, the use of UAV aerial emergency monitoring is becoming increasingly common [31,32,33].

At present, the main problems of ice monitoring in the Yellow River include four aspects. First, there are few field observation sections, especially characteristic sections. Second, there are few field observation times, and it is difficult to obtain ice information. Third, the field observation equipment and technology are not modern, the degree of automation is low, the real-time performance is poor, and the temporal information of ice is discontinuous. Fourth, the field test time is long, and the labor intensity is high. In view of the special monitoring environment of the Yellow River, the trend of field observations of Yellow River ice is expected to lead toward the realization of all-weather and real-time monitoring of the ice thickness, water level, flow ice density, flow ice speed, and other key ice factors. The main problems are discussed in more detail as follows:

(1) There are few field observation sections in the field and few characteristic sections in particular. At present, the ice factor observation data of the Inner Mongolia section of the Yellow River mainly come from the BayanGaole hydrological station, Sanhuhekou hydrological station, Baotou hydrological station, and Toudaoguai hydrological station. This arrangement is still insufficient to fully understand the formation and development of ice jams, ice dams, and ice disasters of the Yellow River, especially at the bend of the river and the river section prone to ice disasters year round.
(2) There are few field observations, and the real-time performance is poor. The existing field observation data are sporadically distributed, some involve only the freeze ice and ice breakup period of a year, and some are in the no-ice period. This makes it difficult to study the ice change law and the ice growth and dissipation change of the Yellow River. In the meantime, the accuracy of the relevant mathematical prediction models is also not high. Therefore, it is necessary to establish a long-term continuous field observation system to obtain the field data of all ice periods of the Yellow River, such as the no-ice period, flow ice period, freeze ice period, and breakup ice period.
(3) The field observation data are not comprehensive. At present, the field data of the ice period of the Yellow River mainly include conventional hydrometeorological data, such as temperature, water temperature, water level, ice thickness, etc. The formation and scale of ice dams and ice jams in ice periods are not only determined by conventional hydrometeorological data, but also related to ice thickness, ice transport velocity, and river section conditions. Other factors should also be considered, such as the amount of sediment transport under ice sheets, the amount of river terrain change, and the amount of channel erosion and deposition. Additional factors, such as air pressure, radiation, sediment concentration, and ice density, should be considered in the mathematical models for predicting the freeze-up time and breakup time. Therefore, it is necessary to observe more influencing factors related to ice in the field and establish a thematic database of ice elements and river terrain databases in the future to provide big data support for the accurate analysis of ice in the Yellow River in the future.
(4) The field observation equipment and technology are not modern, the degree of automation is low, the field test cycle is long, and the labor intensity is high. Field observation equipment and technology are mostly based on artificial methods, which are inefficient and dangerous, especially in periods of ice jams and ice dams. The technology for monitoring frazil ice formation and migration is still lacking. The ice mechanics parameters need to be obtained by indoor experiments.

Therefore, it is necessary to develop new observation equipment for the ice monitoring of the Yellow River, which should be safe, efficient, convenient, and automatic. Therefore, a fixed-point air-coupled radar remote monitoring device is proposed, which provides new ideas and methods for solving the ice monitoring problem in the Yellow River. Many parameters, such as temperature change, flow ice velocity, flow ice density, ice thickness, and water level, have been obtained and analyzed. Furthermore, the effectiveness of the observation results and the advantages and disadvantages of the device are discussed. Finally, a solution to further improve ice observations is proposed. The research results will further improve the ice prevention monitoring capacity of the Yellow River, promote the field observation technology level, increase the accuracy of ice forecast, reduce ice disaster, minimize the loss of people’s lives and property, and improve the public security defense capacity.

## 2. Tests and Methods

### 2.1. System Hardware Composition

**Figure 1:**
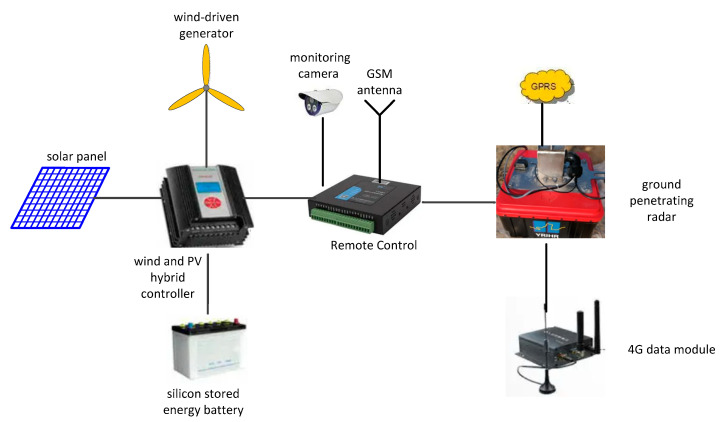
Schematic diagram of the air-coupled radar remote monitoring device system.

The system hardware of the monitoring device mainly includes air-coupled ground penetrating radar, remote switch control and telemetry module, wind and PV hybrid system, GPS module, 4G data transmission module, high-definition infrared night vision instrument (monitoring camera), and other fittings. The components are shown in Figure 1.

Air-coupled ground penetrating radar (GPR) is the core component and is used to detect the ice thickness and the distance between the water surface and the radar. The remote computer sends parameters (sampling points, sampling frequency, accumulation times, acquisition interval, etc.) to the radar through a 4G network and displays, processes, and saves the radar data. After receiving the command, the radar collects the signal and returns the data. The technical parameters are shown in Table 1.

A mobile communication platform (GSM/GPRS network) is used for two-way communication to realize the remote switch control and voltage/temperature control of the system in remote switch control and telemetry modules.

There are two main types of wind and photovoltaic (PV) complementary controllers: Pulse width modulation (PWM) controllers and maximum power point tracking (MPPT) solar controllers. The PWM method is used to control the voltage of the battery in a PWM controller. The method is simple, and the charge efficiency is generally approximately 80%. MPPT controllers can detect the generator voltage of the panel in real time and track the highest current value. They can effectively coordinate the work of solar panels, batteries, and loads.

The GPS module provides a second pulse signal (PPS) and time information for the radar system. According to the PPS signal, the radar timing controls the interval time of radar data acquisition, and the interval time can be set remotely according to the actual needs. The UTC time information provided by GPS is also recorded in the radar return data, and the radar echo data are time tagged in order that the radar data can be traced back according to the time.

A 4G data transmission module is used to realize the long-distance wireless communication between the radar and remote computers.

### 2.2. Installation of Equipment

**Figure 2:**
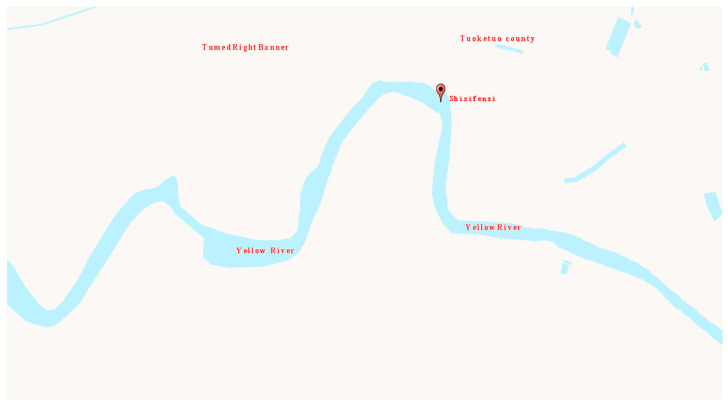
Specific location of equipment installation.

**Figure 3:**
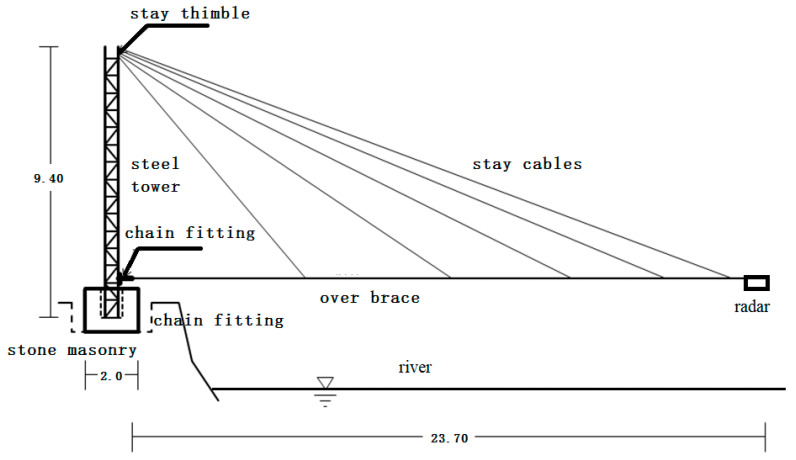
Installation drawing of equipment.

**Figure 4:**
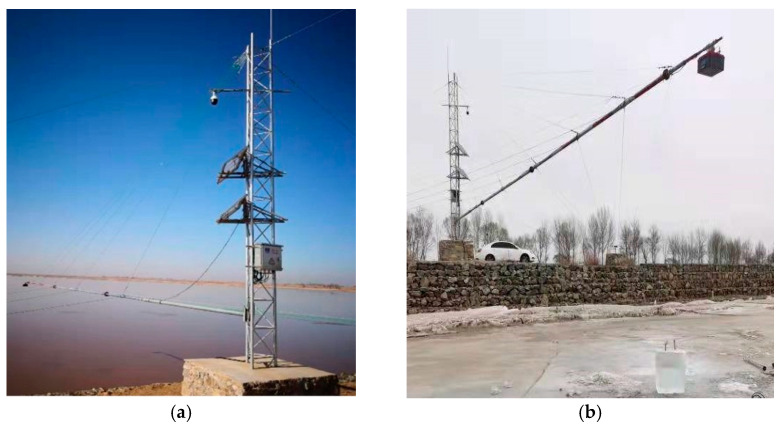
Site drawing of installed equipment. (**a**) Radar equipment from bank view; (**b**) radar equipment from river view.

Through field investigation and analysis, it was determined to install a fixed-point air-coupled radar remote monitoring device for ice thickness and water level in the Shisifenzi reach of the Yellow River in Inner Mongolia. The Shisifenzi observation station is located near the Yellow River embankment at the junction of Tuoketuo County and Tumed Right Banner. This section is one of the typical sections of the Yellow River prone to ice flood disasters. The specific location of this reach is shown in Figure 2. The installation of equipment includes civil engineering aspects, a metal structure and instrument equipment (Figure 3). Among them, the civil engineering aspects include steel tower foundations, fixed column foundations, maintenance platforms, etc. The metal structure includes a steel tower, steel tower embedded parts, installation cross bar, steel tower stay wire (3 pieces), cross bar diagonal stay wire, cross bar side stay wire, fixed column, instrument box, and connector. The instruments and equipment include solar panels, batteries, suspended integrated continuous monitoring devices for ice thickness and water level, control devices, transmission and connecting cables, instrument barrels, etc. The site conditions after installation are shown in Figure 4. The radar is about 5 m above the river surface and the distance varies with the change of river water level.

### 2.3. Experimental Algorithm

When the data are returned by the radar, they are processed and analyzed by the remote computer, enabling the ice thickness and the distance between the radar and the ice surface to be obtained. It is necessary to carry out layer tracking algorithm research on the ice surface and ice water interface and automatically analyze the ice thickness and the distance between the radar and the ice surface through the algorithm. This algorithm mainly uses the reflection wave form and intensity characteristics to capture the changes of the ice water interface and ice/water air interface by tracking the event to calculate the ice cover thickness, water surface, and ice distance from the radar. The basis of the algorithm is to extract the transmitting layer and identify the homogeneity and similarity of reflected waves in the same formation. The two properties are used to track the reflecting layer.

(1) Homogeneity

By comparing of the reflection waves on adjacent frequency channels of the radar spectrum, the lines connected by the same phase of the same reflected wave group on different frequency channels are called the phase axis. The phase characteristics of the same wave group, namely, the position of the wave peak and trough, change slowly along the line.

(2) Similarity

The main characteristics of the same layered reflection wave in the adjacent channel are similar, such as wave form, amplitude, period, and envelope. It is the basis of reflection layer recognition to determine the reflected wave with certain morphological characteristics, and the homogeneity and similarity of reflection waves in the same formation provide the basis for tracking the reflection layer.

The change information of the layer position is automatically tracked based on the detection and tracking method of the Markov model. The continuity of the layer reflection echo is that each measuring point is related only to its adjacent measuring points and not to the other measuring points. Based on the viewpoint of probability and statistics, the Markov model is suitable for this situation. Therefore, it can be used to construct the detection and tracing algorithm of the reflecting layer.

## 3. Results

After the equipment was installed, it operated normally and experienced strong winds, blizzards, low temperatures, heavy fog, and continuously cloudy days. The ice generation and dissipation evolution process of the Shisifenzi reach was observed, including the change in temperature, the change in the flow ice process, the change in ice thickness, and the change in water level.

### 3.1. Temperature Changes

**Figure 5:**
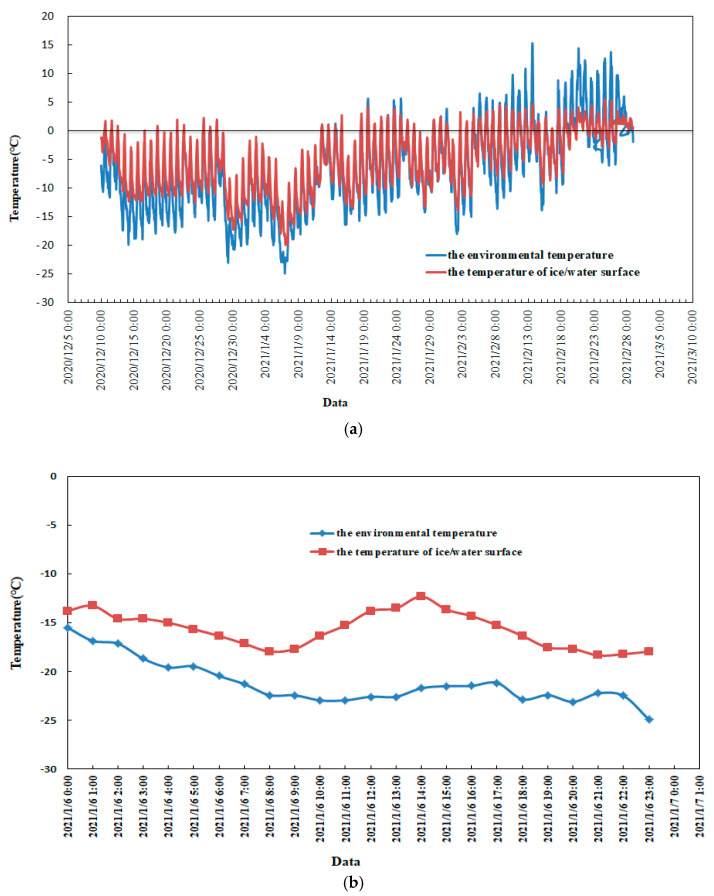
Temperature change process of the Shisifenzi reach. (**a**) Temperature change process of the Shisifenzi reach in 2021–2021 ice period; (**b**) temperature change process of the Shisifenzi reach on 6 January 2021.

Temperature is the main factor affecting the ice situation of the Yellow River. With the decreasing temperature, flow ice appeared in the river, ice gradually increased, and the river was frozen. Thereafter, with the increasing temperature, the ice gradually melted, the river broke up, and the ice disappeared. The temperature change process of the 2020–2021 ice period is shown in Figure 5a. The temperature was monitored every hour, including the environmental temperature and the temperature of the ice/water surface. The minimum environmental temperature was −24.93 °C at 23:00 on 6 January in the 2020–2021 ice period, which is shown in Figure 5b, and the minimum ice temperature was −19.97 °C at 1:00 on 7 January. Figure 5 shows that negative temperatures dominated before mid-February, which directly led to ice formation and river freezing. After mid-February, the positive temperature dominated, and the river gradually melted and broke up.

### 3.2. Flow Ice Velocity and Density

**Figure 6:**
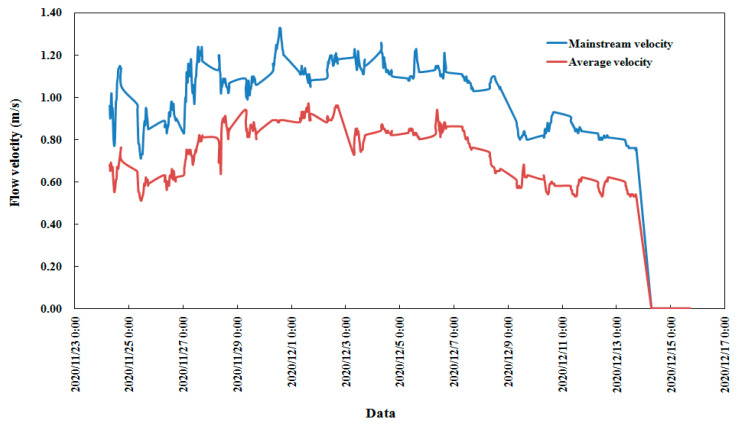
Flow ice velocity of the Shisifenzi reach in the 2020–2021 ice period.

According to the observation and analysis of the test equipment and combined with image recognition technology, the flow ice velocity and density of 2020–2021 were obtained. The evolution process of the flow ice velocity is shown in Figure 6. The first flow ice of the 2020–2021 ice period in the Shisifenzi reach of the Yellow River appeared on 24 November 2020. The river was frozen on 14 December 2020. Two kinds of flow velocities are described in Figure 6: One is the flow ice velocity in the mainstream region of the river, and the other is the average flow ice velocity of the section. The maximum flow ice velocity is 1.33 m/s, which is in the mainstream velocity region. The average flow ice velocity is 0.5–1 m/s. The flow ice velocity was relatively stable during the whole flow ice period of 2020–2021.

**Figure 7:**
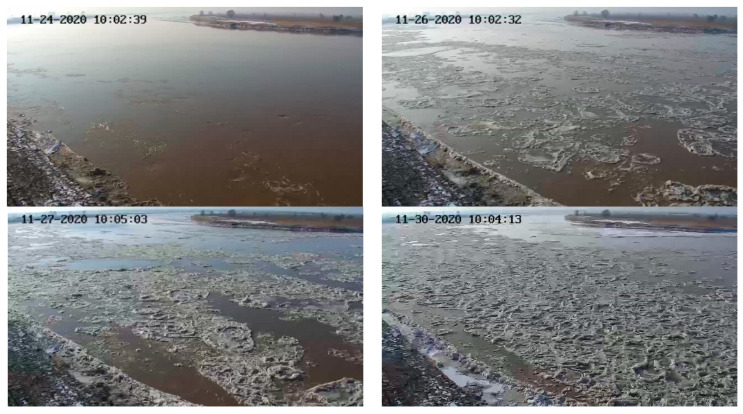

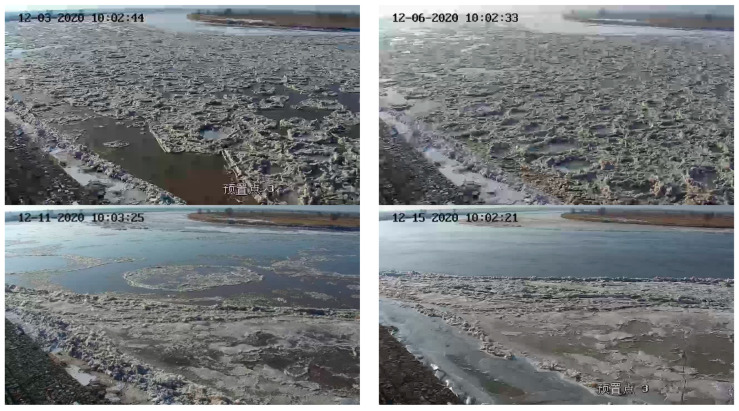
Flow ice scenes on different dates of the 2020–2021 ice period in the Shisifenzi reach.

**Figure 8:**
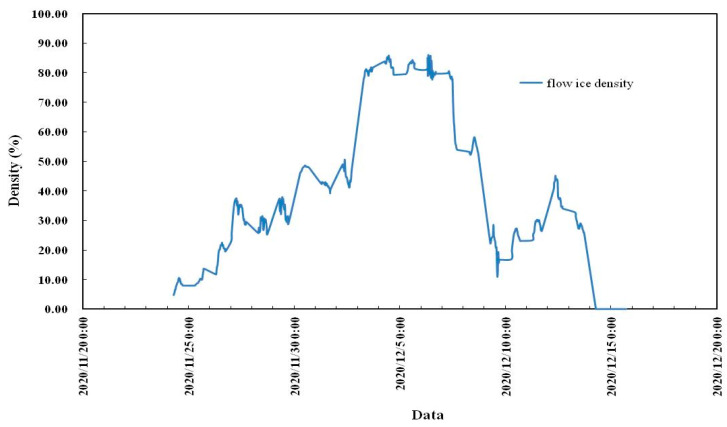
Flow ice density of the Shisifenzi reach in the 2020–2021 ice period.

**Figure 9:**
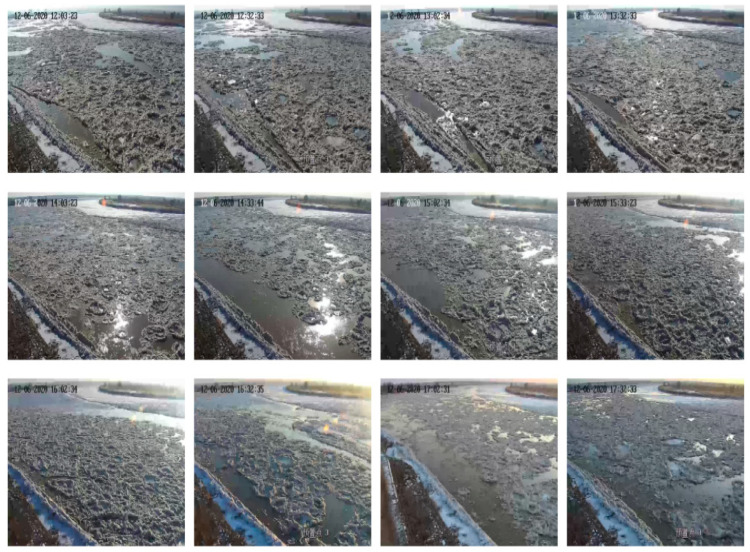
Flow ice scenes in different times of the Shisifenzi reach on 6 December.

Flow ice scenes on different dates of the 2020–2021 ice period in the Shisifenzi reach are shown in Figure 7. The times of different dates in Figure 7 are basically the same, at approximately 10 a.m. The ice growth process of this reach during the 2020–2021 ice period can be understood by comparing the subfigures. To further quantify the flow density, combined with the image processing technology, the evolution process of flow density is shown in Figure 8. The maximum flow ice density appeared on 6 December, which was 85.82%, and then the flow ice density gradually decreased, mainly due to the fact that the ice was basically blocked in this reach and upper reach, and the flow ice in the upper reaches of the river could not be transported to the lower reaches. The flow ice scenes at different times of the Shisifenzi reach in December are shown in Figure 9. On 6 December, the river section was frozen and blocked as a whole.

### 3.3. Ice Thickness and Water Level

**Figure 10:**
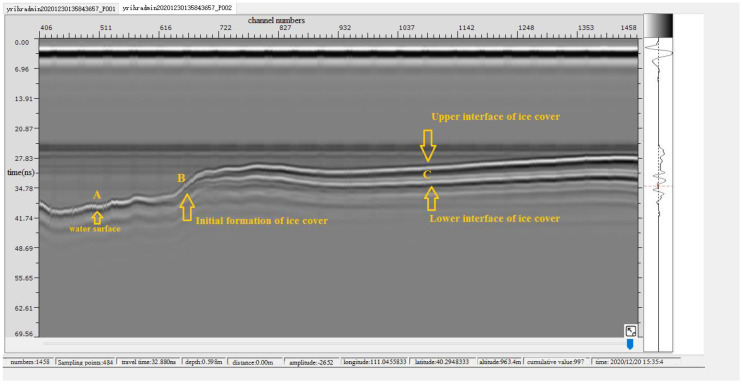
Detected radar spectrums of the Shisifenzi reach in the 2020–2021 ice period.

**Figure 11:**
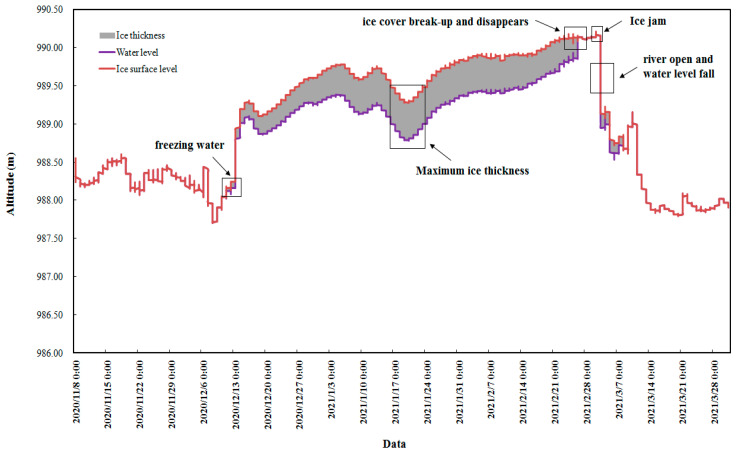
Ice thickness and water level in different times of the Shisifenzi reach in the 2020–2021 ice period.

Based on the suspended air-coupled geological radar technology, ice thickness and water level data were obtained. The change process of ice thickness and water level in the early stage of river ice freezing of the Shisifenzi reach is shown in Figure 10. According to the different characteristics of radar spectrums in different substances, the ice thickness is analyzed and calculated. There was no ice cover formation at the time of A point in Figure 10. Two different radar wave spectrums appeared at the time of point B in Figure 10, indicating that the initial ice cover began to form. Thereafter, the ice thickness gradually developed, and the water level gradually rose. There has been a very obvious upper interface and lower interface of ice cover at C point in Figure 10. The phenomenon is that the evolution of ice thickness and the change process of water level are well described in Figure 11 during the ice periods of 2020–2021. The ice thickness data of this reach were first accurately captured on 11 December 2020. On 18 January 2021, the ice thickness of the river section below the observation point reached the maximum value of 52 cm. On 26 February 2021, the ice cover broke up, floating ice drifted downstream, the ice thickness data disappeared, and the water level decreased significantly, indicating that the river section opened up. From 3 to 8 March 2021, the ice thickness was still measured at the Shisifenzi section, which was mainly caused by floating ice accumulation from upstream.

## 4. Discussion

### 4.1. Verification Analysis of Ice Thickness

**Figure 12:**
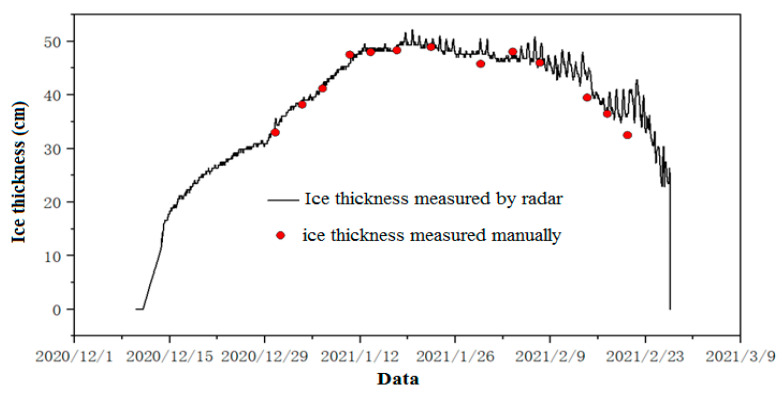
Comparison diagram of ice thickness results measured by radar and manual methods in the 2020–2021 ice period.

To effectively verify the effectiveness and accuracy of the test results of the fixed-point air-coupled radar remote monitoring device, a total of 13 manual holes were drilled at the equipment site to measure the ice thickness in the 2020–2021 ice period. The verification results are shown in Figure 12. The difference range between the ice thickness measured by the equipment and the ice thickness measured manually is 0.001–0.041 m, and the mean error is 0.012 m. The radar monitoring results of the ice thickness are consistent with the manual drilling measurement results during the growth period, while some monitoring results deviate greatly during the ice melting period. This is mainly due to the fact that the ice cover was loose and the dielectric constant changed greatly during the ice melting period. In the follow-up, the dynamic change process of the dielectric constant of the ice cover will continue to be explored during the ice melting period.

### 4.2. Advantages of the Monitoring Device

The device provides fixed-point suspension noncontact integrated continuous monitoring of ice thickness and water level. In addition, it is economical and reliable. Moreover, the device can not only monitor the change process of ice surface elevation, water level under ice, and ice thickness in the ice period, but also monitor the change process of free water surface or ice surface all year round. Furthermore, the device solves four technical difficulties: Air-coupled radar, 25 m long suspension cross bar, integrated solar low-temperature power supply communication, artificial intelligence recognition and identification. The equipment breaks through the technical bottleneck of noncontact and continuous measurement of water level and ice thickness, realizes the real-time monitoring of ice thickness and water level at fixed points, and provides new modes and technologies for hydrological monitoring in ice periods.

### 4.3. Adverse and Supplementary Measures for the Monitoring Device

**Figure 13:**
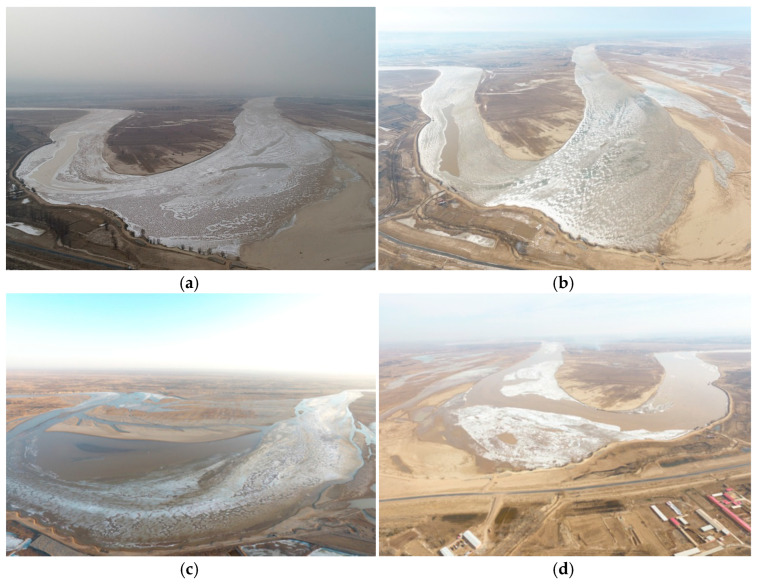
Aerial photographs of the ice generation and disappearance process in the Shisifenzi section during the ice period: (**a**) Ice accumulation and frozen river; (**b**) stable river freeze-up; (**c**) temperature rise and ice cover melting; (**d**) ice breakup and river opening.

Since the equipment is fixed, it can observe only the local ice situation and water changes of the Shisifenzi River section and cannot characterize the river ice changes over a wider range. Next, on the one hand, fixed observation points can be added at other typical sections of the Yellow River. In addition, additional monitoring equipment for ice-related parameters can be added to the system, such as spectrum equipment, ice velocity equipment, and solar radiation monitoring equipment. On the other hand, UAV aerial surveys can be combined with fixed-point monitoring, and the river ice change law can be better characterized. The typical change processes of ice generation and disappearance in the Shisifenzi River section are shown in Figure 13, which were measured by UAV. Figure 13a shows the process of ice accumulation and river freezing, Figure 13b shows the stable freeze-up river period, Figure 13c shows the stage of temperature rise and ice cover melting, and Figure 13d shows the ice breakup and river opening. During the river ice breakup period, the ice cover gradually melted, and the river ice slowly broke up. The macroscopic observation of ice by UAVs and the local microscopic observation of ice by fixed-point equipment can form a better complement.

## 5. Conclusions

It is difficult to effectively monitor the ice change process of the Yellow River in the ice period. A fixed-point air-coupled radar remote monitoring device was proposed in this paper. The research results are expected to contribute to improving the ice monitoring ability of the Yellow River, increasing the field observation technology level, and enhancing the ice prediction accuracy and public security defense ability.

(1) Monitoring technology is a fixed-point, noncontact, and multiparameter ice monitoring technology that can not only synchronously observe the changes in water level and ice thickness, but also observe the ice, water surface temperature, and air temperature, as well as automatically take photos and videos at fixed angles at fixed times and over the whole ice period. The equipment has endured adverse weather conditions, such as blizzards, heavy fog, continuously cloudy days, strong winds above level 10, and a minimum temperature of −31 °C.
(2) The temperature change process in the 2020–2021 ice period was continuously monitored. The minimum environmental temperature was −24.93 °C at 23:00 on 6 January, and the minimum ice temperature was −19.97 °C at 1:00 on 7 January in the 2020–2021 ice period.
(3) The first flow ice of the 2020–2021 ice period in the Shisifenzi reach of the Yellow River appeared on 24 November 2020. The maximum flow ice velocity was 1.33 m/s, which is in the mainstream velocity region. The average flow ice velocity was 0.5–1 m/s. The flow ice velocity was relatively stable during the whole flow ice period of 2020–2021.
(4) The maximum flow ice density occurred on 6 December, which was 85.82%, and then the flow ice density gradually decreased. On 6 December, the river section was frozen and blocked as a whole.
(5) The first ice thickness data were accurately captured on 11 December 2021. The maximum ice thickness reached 52 cm. On 26 February 2021, the ice cover broke up, floating ice drifted downstream, the ice thickness data disappeared, and the water level decreased significantly, indicating that the river section opened up. From 3 to 8 March 2021, ice thickness was still measured at the Shisifenzi section, which was mainly caused by floating ice accumulation from upstream.
(6) Two methods can be adopted to improve the ice monitoring of the Yellow River: One is to add more representative monitoring points, and the other is the combination of local observations and macropatrol surveys.

## Figures and Tables

**Table 1:** Technical parameters of GPR.

Parameters	Values
radar center frequency	400 MHz
sampling points	256/512/1024/2048
sampling frequency	50 GHz/25 GHz/10 GHz/5 GHz
maximum detection time window	400 ns
stacking numbers	1~128
maximum detection thickness	5 m
maximum height of radar from water surface	20 m
power supply mode	hybrid wind/PV power
power consumption	10 W
working temperature	−40~+50 °C
storage temperature	−45~+85 °C
